# The impact of different exercise modalities on health-related quality of life in patients with prostate cancer undergoing androgen deprivation therapy: a systematic review and network meta-analysis

**DOI:** 10.3389/fonc.2026.1821735

**Published:** 2026-08-17

**Authors:** Wang Li, Xiaoqin Huang, Xin Huang, Yiqin Shi, Feng Wang, Liping Pu, Mingyan He, Huiwen Tan

**Affiliations:** 1 Department of Urology, The Second Affiliated Hospital of Chongqing Medical University, Chongqing, China; 2 Acute and Critical Care Nursing Innovation and Translation Engineering Research Center of Chongqing Education Commission of China, Chongqing, China; 3 Chongqing Geriatric Specialized Nursing Clinical Diagnosis and Treatment Center, Chongqing, China

**Keywords:** androgen deprivation therapy, exercise, health-related quality of life, network meta-analysis, prostate cancer

## Abstract

**Background:**

Androgen deprivation therapy (ADT) for prostate cancer is associated with adverse effects including fatigue, muscle weakness, adiposity, and impaired health-related quality of life (HRQoL). Exercise interventions may mitigate these side effects, but the comparative efficacy of different exercise modalities remains unclear.

**Purpose:**

To determine the effects of various exercise programs on HRQoL and physical functioning in prostate cancer patients undergoing ADT, and to provide evidence-based recommendations for clinical practice.

**Methods:**

We systematically searched Chinese and English databases (PubMed, Embase, Cochrane Library) from inception to May 2024 for randomized controlled trials. Two independent reviewers performed screening, data extraction, and risk of bias assessment. A network meta-analysis was conducted using StATA 17.0, with mean differences (MDs) and 95% confidence intervals (CI) as effect measures. Interventions were ranked using the surface under the cumulative ranking curve (SUCRA).

**Findings:**

41 RCTs were found in total and comprised 3, 588 participants. Combined aerobic and resistance exercise (AE+RE) was most effective for improving global HRQoL (FACT-P: MD = 7.96, 95% CI: 2.95–12.96). Resistance exercise (RE) ranked highest for increasing lean body mass (MD = 2.79, 95% CI: 0.68–6.31) and muscle strength (lower limbs: MD = 38.03; upper limbs: MD = 6.71). AE+RE was most effective for exercise capacity (6-minute walk test: MD = 51.10). For fatigue reduction, low-to-moderate intensity exercise ranked highest, though between-intervention differences were non-significant.

**Conclusion:**

AE+RE is the optimal intervention for improving overall HRQoL and exercise capacity in ADT patients. RE confers superior benefits for muscle strength, while AE is more effective for reducing adiposity. A combined exercise regimen should be recommended as standard care, with individualization based on patient needs. Long-term follow-up studies are warranted.

**Systematic Review Registration:**

https://www.crd.york.ac.uk/prospero/display_record.php?RecordID=420250408942, identifier CRD420250408942.

## 1 Introduction

Prostate cancer (PCa) is the second most common cancer among men worldwide, with its incidence continuing to rise in both developed and developing countries, and it represents one of the leading causes of cancer-related mortality in males (1, 2). According to the 2022 data from the Global Cancer Observatory (GLOBOCAN), there were approximately 1.47 million new cases globally (accounting for 14.2% of all male cancers) and about 397, 000 deaths (representing 7.3% of male cancer-related mortality) (2). The incidence of prostate cancer increases significantly with age, and men aged 65 years and older are widely considered a high-risk population. In addition, environmental factors such as dietary patterns and lifestyle have been recognized as relevant to its risk of development (3, 4).

Currently, the main treatment strategies for prostate cancer include active surveillance, radical prostatectomy, radiation therapy, and androgen deprivation therapy (ADT). Among these, radical prostatectomy and radiation therapy serve as the primary treatments for localized prostate cancer, albeit with a risk of recurrence. ADT, as the first-line treatment for metastatic prostate cancer (5), is primarily achieved through surgical castration or medical castration (using gonadotropin-releasing hormone agonists or antagonists) (6). ADT has become a standard therapeutic option across various clinical scenarios in prostate cancer management, whether used alone or in combination with other treatments (7), and a substantial proportion of patients receive it during the course of their disease (8, 9).

While androgen deprivation therapy (ADT) demonstrates significant efficacy in managing prostate cancer, its adverse effects should not be overlooked. By suppressing androgen levels, ADT may result in reduced muscle mass and impaired physical function. Patients treated with ADT experience significant changes in body composition, including increased fat, loss of lean body mass, and reduced muscle mass (10, 11). Studies indicate that even after accounting for increases in fat mass, patients still exhibit impairments in muscle quality, volume, and strength (12). ADT-induced low serum testosterone directly compromises body composition and muscle strength, leading to a decline in physical function and exacerbation of fatigue. This fatigue further limits physical activity, thereby establishing a vicious cycle (13), These changes not only negatively impact patients’ daily activity capacity and quality of life, which includes reduced energy and emotional distress (14, 15), but may also increase the risk of adverse cardiovascular events due to muscle loss and increased subcutaneous fat (16).

Androgen deprivation therapy (ADT) can also lead to decreased bone mineral density, osteoporosis, and a significantly increased risk of fragility fractures. Reduced testosterone levels accelerate bone loss, a mechanism primarily attributed to impaired bone remodeling caused by declining sex hormones, while mechanical alterations and endocrine consequences resulting from muscle loss also play an indirect role (17). Patients receiving long-term ADT show markedly reduced bone mineral density, particularly at the hip and spine, making fractures a major health threat in this population. A retrospective study indicated that the fracture risk in ADT-treated patients is 1.39 times higher than in those not receiving ADT (18). Thus, ADT accelerates bone loss in prostate cancer patients, induces osteoporosis, and increases fracture risk. Moreover, ADT patients with a history of fractures have a higher overall mortality (19), which significantly compromises their health-related quality of life (HRQoL) and life expectancy.

In addition, ADT can also induce cognitive health issues such as depression, anxiety, and cognitive decline (20–22) which may be related to rapid hormonal changes, reduced physical strength, and decreased quality of life (22). These side effects collectively pose serious challenges to patients’ physical function and HRQoL. As a multidimensional concept, HRQoL reflects patients’ self-perceived impact of the disease and its treatment on their physical, psychological, and social well-being (23). It plays a crucial role in the management of ADT patients, emphasizing not only survival time but also quality of life and overall well-being (24). Therefore, HRQoL assessment is particularly critical for patients undergoing ADT. The 2018 International Multidisciplinary Consensus Roundtable recommended exercise intervention during and after treatment as a standard strategy to alleviate various adverse effects, including physical functional decline, fatigue, mental health issues, and reduced HRQoL (25). Exercise intervention can mitigate ADT-related side effects by enhancing muscle mass and strength, improving cardiorespiratory fitness, optimizing physical function, and promoting psychosocial well-being, thereby improving patients’ physical function and HRQoL. Strong evidence indicates that exercise therapy can address issues such as muscle loss, fatigue, and decreased physical function caused by ADT (26, 27). Although most forms of exercise yield positive benefits, the optimal exercise regimen remains unclear, and systematic evaluation is still needed to compare the effects of different exercise modalities on ADT patients.

Network meta-analysis quantifies multiple interventions and prioritizes their significance within the network. This study therefore employs network meta-analysis to compare the effects of different exercise regimens on health-related quality of life in prostate cancer patients undergoing androgen deprivation therapy (ADT), aiming to provide guidance for selecting optimal exercise modalities.

## 2 Method

This study strictly followed the Preferred Reporting Items for Systematic Reviews and Network Meta-Analyses (PRISMA-NMA) guidelines, aiming to systematically evaluate the effects of different exercise interventions on health-related quality of life (HRQoL) and physical function in prostate cancer patients undergoing androgen deprivation therapy (ADT).This systematic review was conducted in accordance with the Preferred Reporting Items for Systematic Reviews and Meta Analyses (PRISMA) statement and was registered in the PROSPERO database under the registration number CRD420250408942.

No artificial intelligence (AI) tools were used for data analysis, interpretation, or manuscript drafting in this study. All screening, data extraction, and writing were performed manually by the researchers.

### 2.1 Search strategy

The following Chinese and English databases were systematically searched: PubMed, Embase, Cochrane Library, Web of Science, CINAHL, China National Knowledge Infrastructure (CNKI), Wanfang Data, and VIP Chinese Journal Full-text Database. The search time range covered from the establishment of each database to May 2024. The search strategy combined subject terms with free words, with English search terms including: “prostate cancer”, “androgen deprivation therapy”, “exercise”, “aerobic training”, “resistance training”, “quality of life”, “fatigue”, etc.; Chinese search terms included: “prostate cancer”, “androgen deprivation therapy”, “exercise intervention”, “aerobic exercise”, “resistance training”, “quality of life”, “fatigue”, etc. The specific search strategy was developed with the participation of information science experts to ensure the comprehensiveness and accuracy of the search.Detailed search strategies for each database are available in the **Supplementary Table 2**.

### 2.2 Study selection and eligibility criteria

The study included the following criteria:

1. Study Type: Randomized Controlled Trial (RCT);
2. Study subjects: Adult male patients with pathologically confirmed prostate cancer who are currently receiving androgen deprivation therapy (ADT)
3. Intervention: Any form of exercise intervention (e.g. aerobic exercise alone, resistance training, aerobic combined resistance training, high-intensity interval training, etc.), the control group was routine nursing, placebo intervention or no exercise intervention, with an intervention duration of ≥ 4 weeks;
4. Outcomes: The primary outcome was health-related quality of life (HRQoL), assessed using scales such as FACT-P, EORTC QLQ-C30, or SF-36. Secondary outcomes included body composition (lean body mass, body fat mass, body fat percentage), muscle strength (upper limbs, lower limbs), physical fitness (400-meter walk test, 6-minute walk test, VO_2_peak), and fatigue (FACT-F);
5. Language restriction: Chinese and English

The exclusion criteria were as follows:

1. Non-randomized designs (e.g., observational studies, reviews, case reports);
2. Patients not diagnosed with prostate cancer, not receiving androgen deprivation therapy (ADT), or mixed cancer populations without separate data;
3. Interventions: no exercise component; combined with other non-exercise interventions (e.g., diet, drugs); exercise modalities not fitting predefined categories (aerobic, resistance, combined, HIIT, low-intensity); duration <4 weeks;
4. Control groups that included any exercise or lacked a non-exercise comparator;
5. Health-related quality of life (HRQoL) not assessed with validated instruments (FACT-P, EORTC QLQ-C30, SF-36) or data insufficient for effect size calculation;
6. Non-English/Chinese publications, duplicate reports, or full text unavailable.

### 2.3 Data extraction and quality evaluation

Two researchers (LW and SYQ) independently extracted data from self-designed statistical tables. LW was responsible for screening studies from PubMed, Embase, and Cochrane Library, while SYQ handled Web of Science, CINAHL, and Chinese databases (CNKI, Wanfang, VIP)., seeking assistance from a third researcher when necessary. The extracted data primarily included: ① General information: such as the first author’s name and publication year; ② Research characteristics: design type, sample size of each group, age, etc.; ③ Intervention measures: exercise type, intensity, intervention duration; ④ Outcome indicators: relevant pre-intervention and post-intervention statistical data or *post-hoc* values for estimating effect size, along with the assessment tools used. All screening and full-text reading were performed manually by the researchers (LW and SYQ), not by AI tools.

For outcome measures, the following operational definitions were applied: Upper limb strength was measured using bench press or handgrip dynamometry (kg), while lower limb strength was assessed using leg press or knee extension tests (kg or N). Body fat mass (BFM) refers to the absolute amount of fat tissue in the body (measured in kg), while body fat rate (BFR) represents the proportion of fat mass relative to total body weight (expressed as a percentage). These two metrics provide complementary information and should not be used interchangeably.

The methodological quality of the included randomized controlled trials (RCTs) was assessed using the Cochrane Systematic Reviews Manual 5.1’s bias risk assessment tool for RCTs, evaluating the following aspects: ① randomization sequence generation; ② concealed assignment; ③ blinding of both investigators and participants; ④ blinding of outcome assessors; ⑤ completeness of outcome data; ⑥ selective reporting; ⑦ other biases. Each criterion was rated as high risk, low risk, or uncertain risk. Researchers interpreted and evaluated the quality based on the descriptive details of the included studies.

### 2.4 Statistical processing

When at least two studies are available, a paired meta-analysis is performed to compare the effects of one exercise type versus another or a control condition. Using a random effects model, the mean difference (MD) and 95% confidence interval (95%CI) are calculated. Heterogeneity is classified as very low (I2<25%), low (I2 = 25-50%), moderate (I2 = 50-75%), or high (I2≥75%) based on the I2 statistic.

The paired (pairwise) meta-analysis calculates effect sizes based only on direct comparisons between two interventions (e.g., AE vs. control), using all available studies that compare these specific interventions. In contrast, the network meta-analysis (NMA) simultaneously analyzes both direct comparisons (when two interventions are directly compared in studies) and indirect comparisons (when interventions share a common comparator, such as control). The NMA effect sizes incorporate information from the entire network of interventions, potentially providing more precise estimates and allowing ranking of all interventions even when head-to-head comparisons are lacking. For readers unfamiliar with these methods: an open-loop network refers to interventions connected only through indirect comparisons without forming closed loops, while a closed-loop network contains triangular or higher-order loops where multiple interventions are directly compared, enabling assessment of consistency between direct and indirect evidence (node-splitting analysis).

The consistency test was conducted using the node splitting model, where a P-value <0.05 indicates a statistically significant difference between direct and indirect comparison results. Network relationship diagrams and rank plots were constructed for each intervention measure. The cumulative rank probability area (SUCRA) was used to rank the interventions, with larger areas indicating better efficacy. A funnel plot was created to identify potential publication bias. Meta-analysis was performed using the Meta package in Stata17.0 software.

## 3 Result

### 3.1 Results and characteristics of the literature search

**Figure 1:**
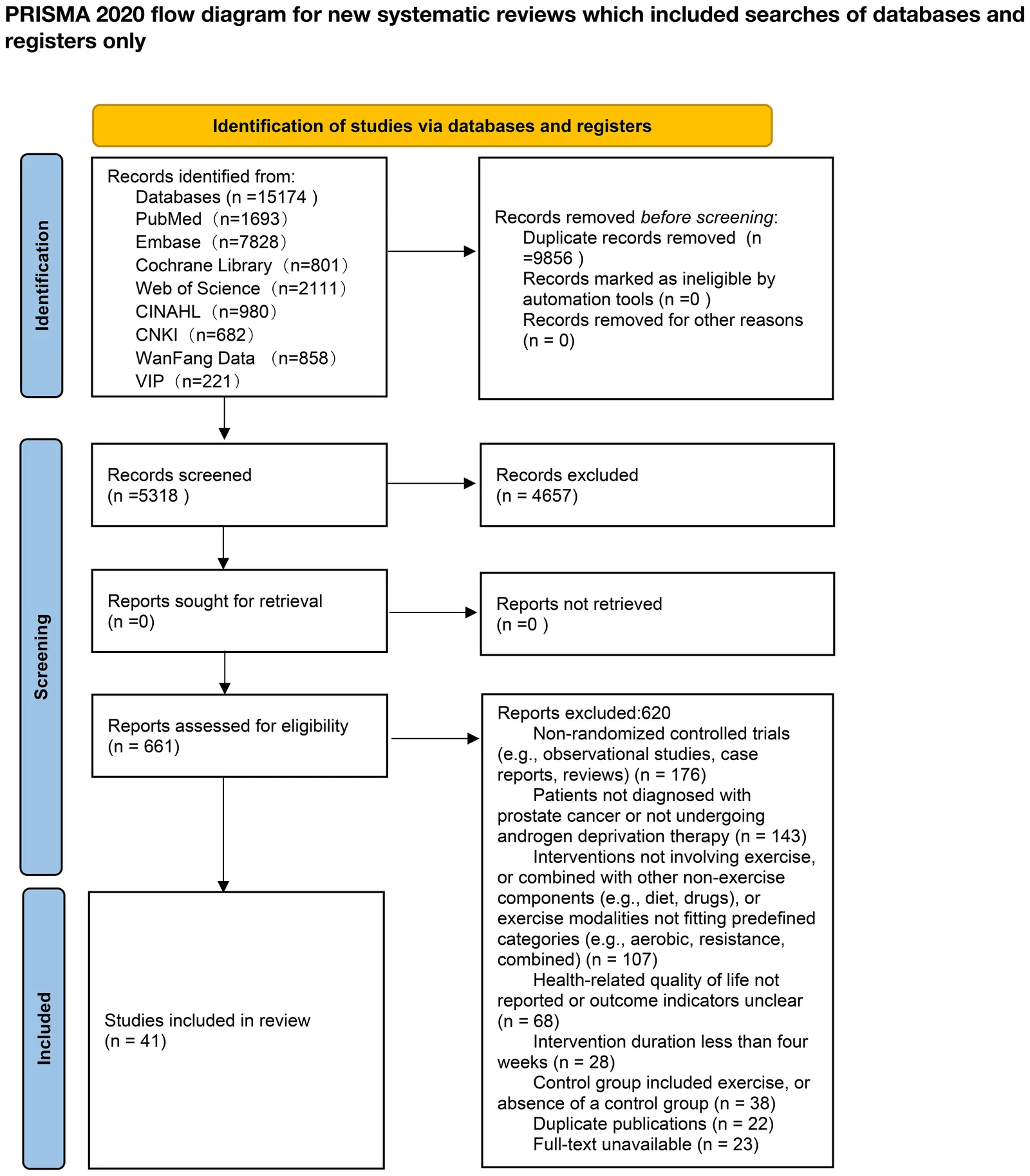
PRISMA flow diagram of study selection process for the impact of different exercise modalities on health-related quality oflife in patients with prostate cancer undergoing androgen deprivation therapy

Through systematic retrieval of Chinese and English databases such as PubMed, Embase, Cochrane Library, Web of Science, CINAHL, China National Knowledge Infrastructure (CNKI), Wanfang Database, and VIP Chinese Journal Full-text Database, a total of 15174 related literature records were initially obtained. After deduplication using EndNote 20 software, 5318 articles remained. By reviewing titles and abstracts, 4657 articles that did not meet the inclusion criteria were excluded, leaving 661 articles for full-text screening. After full-text reading, 620 articles that did not meet the standards or had incomplete data were excluded, ultimately including 41 randomized controlled trials (RCTs) involving 3, 588 prostate cancer patients receiving androgen deprivation therapy (ADT). The literature screening process strictly followed the PRISMA guidelines, with detailed procedures shown in **Figure 1**.

The 41 studies included spanned from 2003 to 2025, with participants from multiple countries including Australia, Canada, the United States, the United Kingdom, Denmark, Poland, and Belgium. Intervention durations ranged from 8 to 96 weeks. Exercise interventions included aerobic exercise (AE), resistance training (RE), aerobic-resistance combined training (AE+RE), high-intensity interval training (HIIT), HIIT combined with resistance training (HIIT+RE), low-intensity exercise (LMI), and qigong/tai chi. The control groups primarily received routine care, waiting list controls, or stretching-relaxation training.

### 3.2 Risk of bias assessment

**Figure 2:**
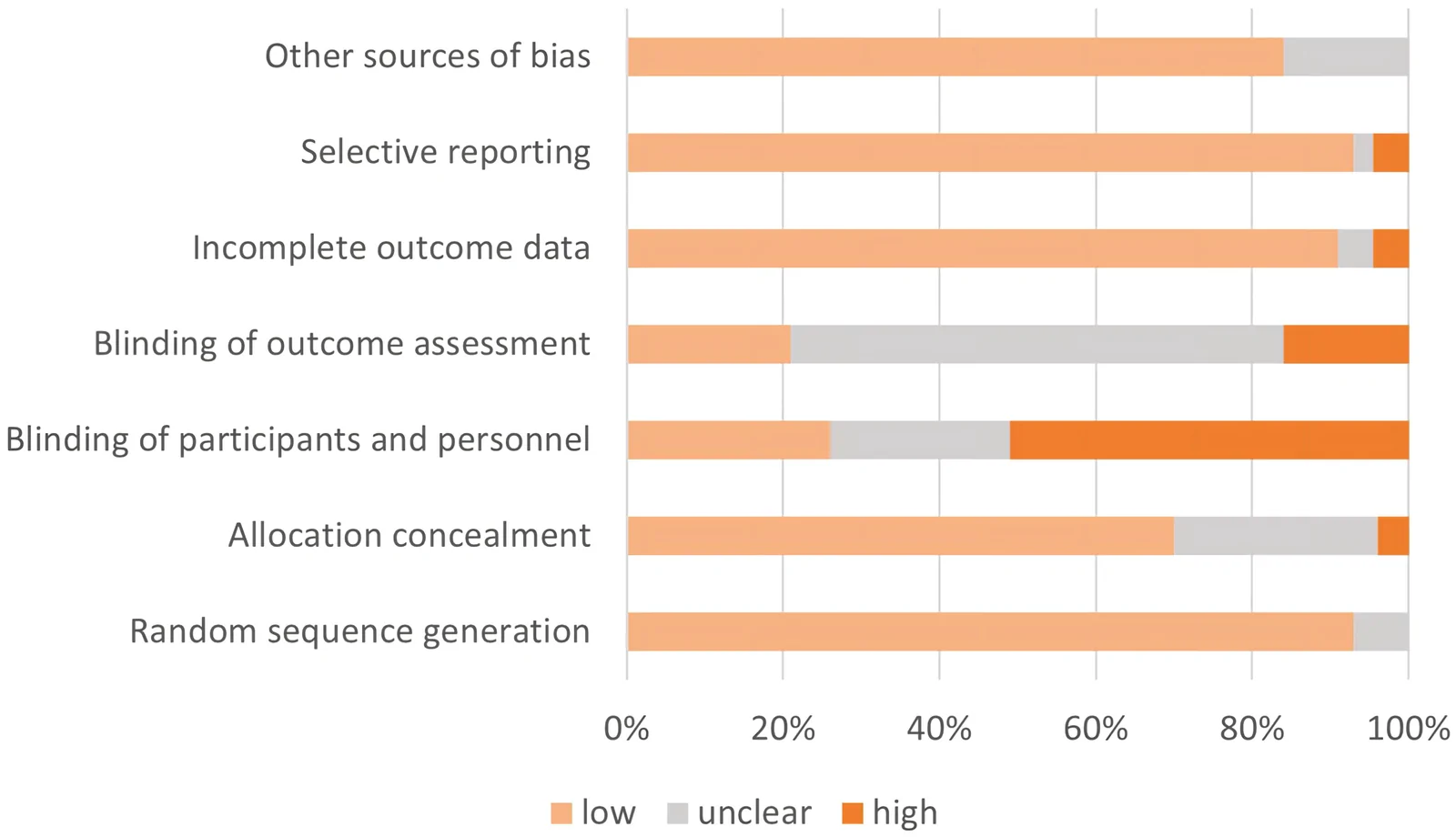
Risk of Bias Assessment: review authors‘ judgments about each risk of bias item for each included study.

The results of the Risk of Bias (RoB) assessment are as follows: (1) Random sequence generation (unclear risk: 6.98%, low risk: 93.02%); (2) Concealed allocation (high risk: 4.65%, unclear risk: 25.58%, low risk: 69.77%); (3) Blinded participants and staff (high risk: 51.16%, unclear risk: 23.26%, low risk: 25.58%); (4) Blinded assessment of outcomes (high risk: 16.28%, unclear risk: 62.79%, low risk: 20.93%); (5) Incomplete outcome data (high risk: 4.65%, unclear risk: 4.65%, low risk: 90.70%); (6) Selective reporting (high risk: 4.65%, unclear risk: 2.33%, low risk: 93.02%); (7) Other bias sources (unclear risk: 16.28%, low risk: 83.72%). Among the 41 included articles, 4 were classified as having high bias risk. The bias risk assessment of the included literature is shown in **Figure 2**. These high-risk studies were retained in the analysis, but their influence was evaluated through sensitivity analyses. The main conclusions remained robust after excluding these studies.

### 3.3 Effect of exercise on quality of life (FACT-P)

Two studies directly investigated the treatment effects of AE and control group on quality of life (FACT-P), two studies directly investigated the treatment effects of RE and control group on quality of life (FACT-P), and six studies directly investigated the treatment effects of AE+RE and control group on quality of life (FACT-P).

Paired meta-analysis showed that AE and control group (MD, 1.56; 95%CI-3.24-6.35), RE and control group (MD, 2.60; 95%CI-1.11, 6.31), AE+RE and control group (MD, 8.08; 95%CI 3.43, 12.95).

**Figure 3:**
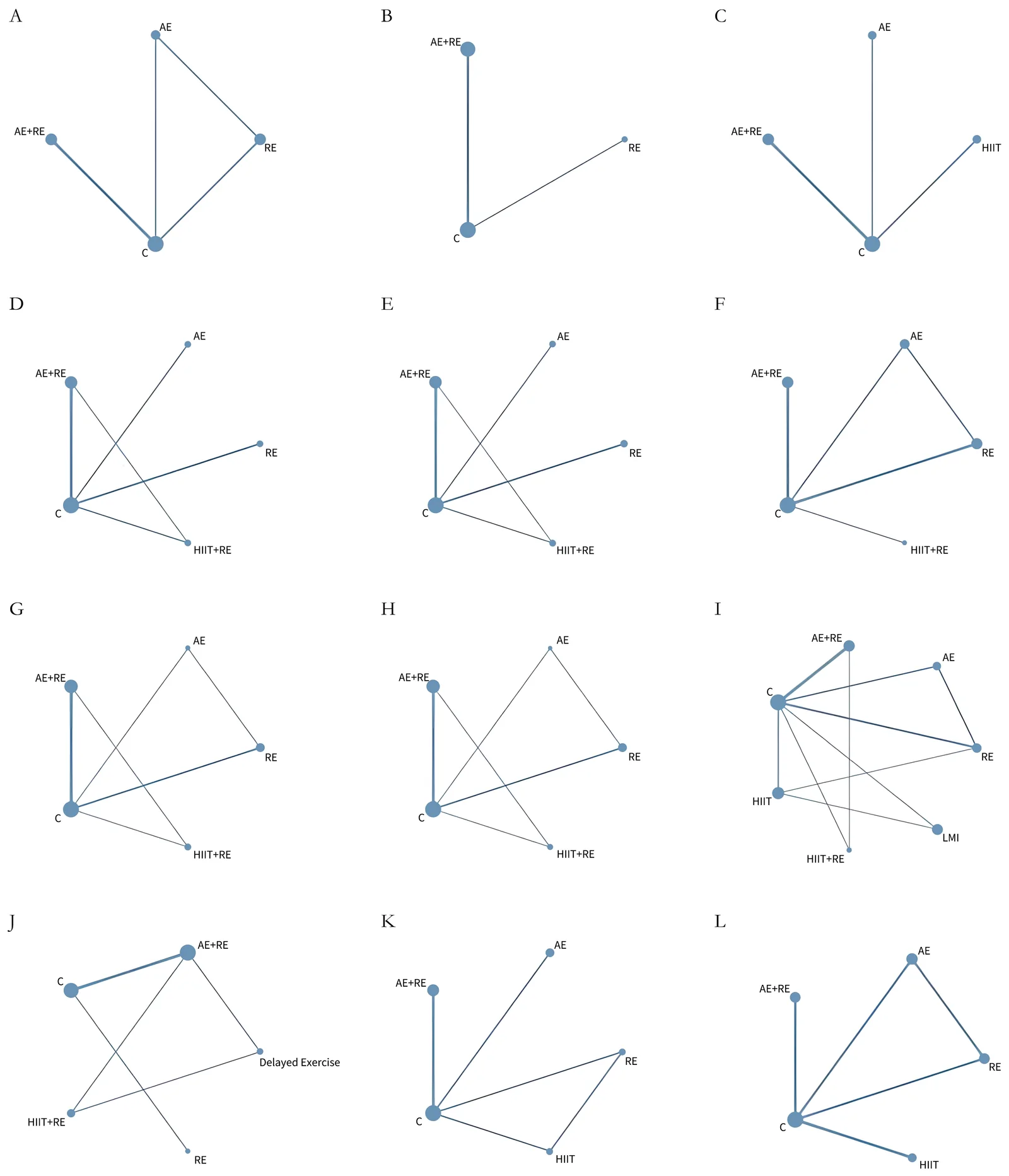
Presents a network meta-analysis comparing: **(A)** Quality of Life (FACT-P), **(B)**Quality of Life (EORTC QLQ-C30), **(C)** Quality of Life (SF-36), **(D)** Lean Body Mass (LBM), **(E)** Body Fat Mass (BFM), **(F)** Upper Limb Strength, **(H)** Lower Limb Strength, **(I)** Fatigue (FACT-F), **(J)** 400-meter walk test, **(K)** 6-minute walk test (m), and **(L)** VO2peak. The network diagram, generated through meta-analysis, features intervention types as nodes and direct comparison studies as lines. Node sizes reflect participant numbers in specific interventions, while line thickness indicates the number of randomized controlled trials.

**Figure 4:**
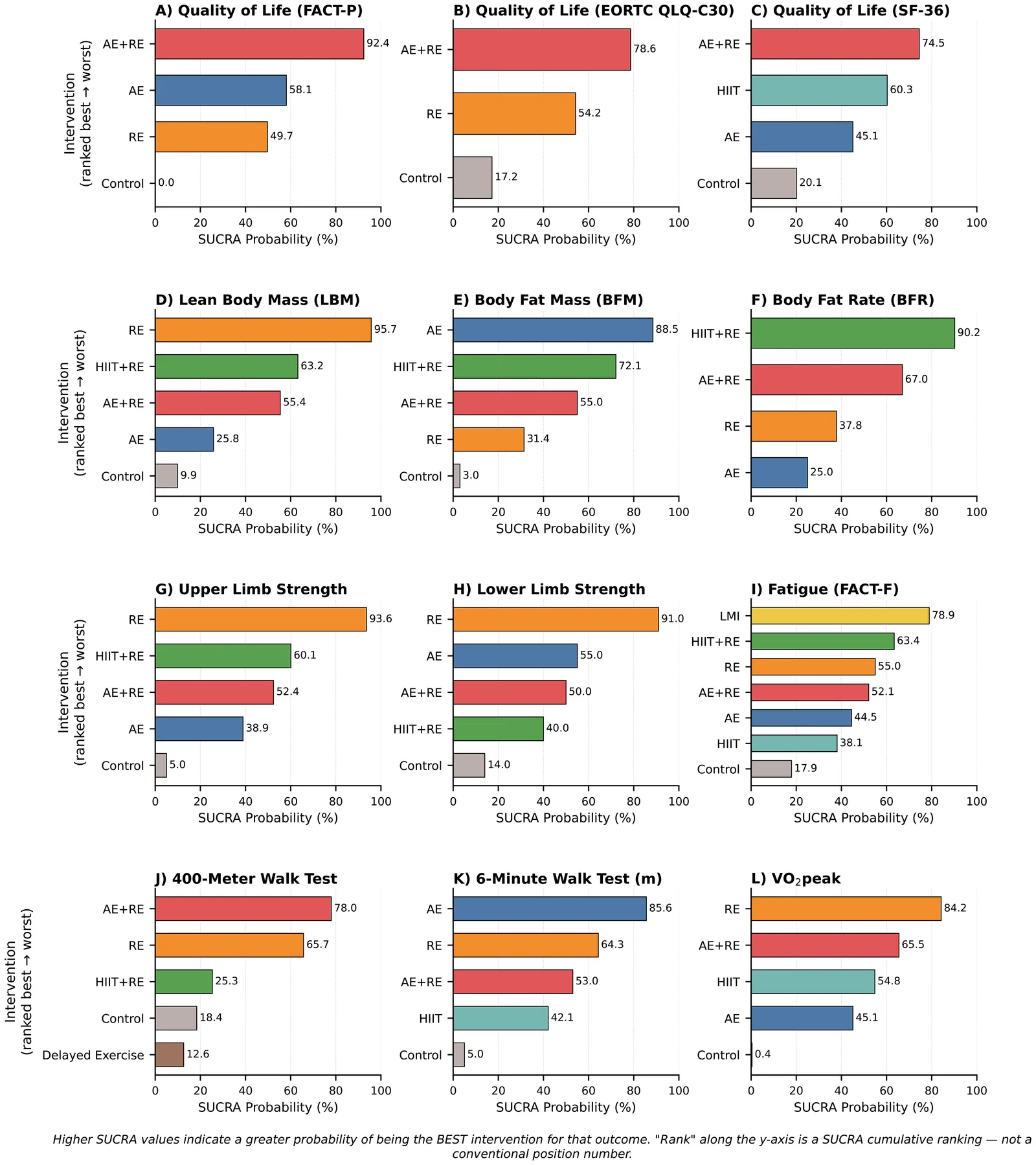
SUCRA (surface under the cumulative ranking curve) values (0 -100%) for each exercise intervention across 12 outcomes: **(A)** FACT_P, **(B)** EORTC QLQ_C30, **(C)** SF_36, **(D)** Lean Body Mass, **(E)** Body Fat Mass, **(F)** Body Fat Rate, **(G)** Upper Limb Strength, **(H)** Lower Limb Strength, **(I)** Fatigue (FACT_F), **(J)** 400_meter walk test, **(K)** 6_minute walk test, **(L)** VO_2_peak. Panel **(F)** has been corrected from Body Fat Mass (BFM) to Body Fat Rate (BFR).

The network analysis included 10 articles and 806 participants. As node segmentation analysis showed no significant inconsistency (all p-values>0.05), a network meta-analysis based on the consistency model was conducted. The results of the network analysis revealed that the AE+RE intervention was superior to the control group (MD: 7.96; 95% CI: 2.95–12.96), while other comparisons were not statistically significant (**Figure 3A**). According to the probability ranking, AE+RE was most likely to rank highest, followed by AE and RE (**Figure 4A**).

### 3.4 Effect of exercise on quality of life (EORTC QLQ-C30)

Five studies directly compared AE+RE versus control group in quality of life (EORTC QLQ-C30). A meta-analysis of these studies showed that AE+RE resulted in a mean difference of 2.57 (95% CI: -2.45 to 7.60) in quality of life.

The network analysis included 6 articles and 430 participants. As the closed-loop was not constructed in the quality of life (EORTC QLQ-C30) meta-analysis, node splitting analysis was not applicable. The consistency model-based meta-analysis showed no significant difference between RE and AE+RE (**Figure 3B**). The ranking of exercise interventions indicated that AE+RE was the most likely to be the most effective form of exercise for improving quality of life (EORTC QLQ-C30) (**Figure 4B**).

The different number of studies across outcome measures (e.g., EORTC QLQ-C30 vs. SF-36) reflects variations in how frequently these instruments were used in the included trials. EORTC QLQ-C30 was the most commonly employed HRQoL measure in exercise oncology trials, while SF-36 was used less frequently, resulting in fewer eligible studies for network analysis.

### 3.5 Effect of exercise on of quality of life (SF-36)

Three studies directly investigated the treatment effects of AE+RE versus control group on quality of life (SF-36). A paired meta-analysis showed that AE+RE had a significant difference from control group (MD, 2.19; 95%CI0.28 to 2.76).

The network analysis included 5 articles and 370 participants. As the closed-loop design was not established in the quality of life (SF-36) network meta-analysis, node splitting analysis was not applicable. The consistency model-based network meta-analysis revealed no significant differences between AE, HIIT, and AE+RE (**Figure 3C**). The ranking of exercise interventions indicated that AE+RE was most likely to rank highest, followed by HIIT (**Figure 4C**).

### 3.6 Effect of exercise on lean body mass

Two studies directly compared AE versus control in treating Lean Body Mass (LBM), four studies compared RE versus control, two studies compared HITT+RE versus control, and eight studies compared AE+RE versus control.

Paired meta-analysis revealed statistically significant differences: RE showed a mean difference (MD) of 2.79 (95%CI 0.68-6.31) compared to the control group (MD = 0.89; 95%CI 0.01-1.77) when AE+RE was added. AE alone (MD = -1.00; 95% CI-4.43 to 2.43) and HITT+RE (MD = -0.03; 95% CI-2.92 to 2.86) showed no significant differences from the control group.

The network analysis included 15 articles and 966 participants. As node segmentation analysis showed no significant inconsistency (all p-values>0.05), a network meta-analysis based on the consistency model was conducted. The results revealed that RE intervention outperformed AE (MD, 3.65; 95%CI 0.89; 6.42), while AE+RE was superior to control (MD, 0.92; 95%CI 0.04; 1.80). RE outperformed control (MD, 2.79; 95%CI 0.68; 4.91), but other comparisons were statistically insignificant (**Figure 3D**). According to the ranking probability, RE was most likely to rank highest, followed by HIIT+RE and AE+RE (**Figure 4D**).

### 3.7 Effect of exercise on body fat mass

Among 18 studies on Body Fat Mass (BFM), two directly compared AE with control group efficacy, five compared RE with control group, two compared HIIT+RE with control group, and ten compared AE+RE with control group.

Paired meta-analysis revealed significant differences in three comparisons: AE versus control group (MD, -3.30; 95%CI-5.57, -0.84), HITT+RE versus control group (MD, -3.76; 95%CI-6.42, -1.11), and AE+RE versus control group (MD, -1.13; 95%CI-2.24, -0.02). No significant difference was found between RE and control group (MD, -0.16; 95%CI-2.21, 1.90).

Consistency model-based meta-analysis revealed that both AE and AE+RE improved Body Fat Mass (BFM) (**Figure 3E**). The probability rankings of all exercise interventions in BFM improvement (best to worst) were: AE, HITT+RE, AE+RE, and RE (**Figure 4E**).

### 3.8 Effect of exercise on body fat rate

Among 14 studies on Body Fat Rate (BFR), two directly compared AE with control group for Body Fat Mass (BFM), five compared RE with control group, and six compared AE+RE with control group.

Paired meta-analysis showed no significant difference between AE+RE and the control group (MD, -1.30; 95%CI-2.07, -0.52), AE and the control group (MD, -1.67; 95%CI-3.58, 0.25), or RE and the control group (MD, -0.42; 95%CI-0.92, 0.12).

Since the node segmentation analysis showed no significant inconsistency (all p-values>0.05), a network meta-analysis based on the consistency model was conducted. The results demonstrated that HITT+RE outperformed RE in improving Body Fat Rate (BFR) (MD, -3.48; 95%CI, -6.70 to-0.27), HITT+RE surpassed AE in BFR improvement (MD, -3.81; 95%CI, -7.08 to-0.53), and AE+RE was superior to AE in BFR enhancement (MD, -1.07; 95%CI, -2.12 to-0.03) (**Figure 3F**). The ranking probability of all exercise interventions in improving Body Fat Mass (BFM) (from best to worst) was: HITT+RE, AE+RE, RE, and AE (**Figure 4F**).

### 3.9 Effect of exercise on upper limb strength

Four studies directly compared RE with control groups in upper limb strength, while nine studies compared AE+RE with control groups.

Paired meta-analysis showed that RE and control group (MD, 6.33; 95%CI 3.36, 9.31) and AE+RE and control group (MD, 3.6; 95%CI 1.26-5.94).

The network analysis included 13 articles and 928 participants. As node segmentation analysis showed no significant inconsistency (all p-values>0.05), a network meta-analysis based on the consistency model was conducted. The results revealed that RE intervention outperformed the control group (MD: 6.71; 95%CI: 2.84–10.59), while AE+RE showed superiority over the control (MD: 3.62; 95%CI: 1.23–6.00). Other comparisons were statistically insignificant (**Figure 3G**). According to the probability ranking, RE was most likely to rank highest, followed by HIIT+RE, AE+RE, and AE (**Figure 4G**).

### 3.10 Effect of exercise on of lower limb strength

Four studies directly compared RE with control groups in upper limb strength, while ten studies directly compared AE+RE with control groups.

Paired meta-analysis showed that RE and control group (MD, 56.1; 95%CI-8.56, 120.77) and AE+RE and control group (MD, 18.64; 95%CI 11.87, 25.42).

The network analysis included 14 articles and 981 participants. As node segmentation analysis showed no significant inconsistency (all p-values>0.05), a network meta-analysis based on the consistency model was conducted. The results of the network analysis revealed that RE intervention was superior to the control (MD: 38.03; 95% CI: 16.95; 59.12), while other comparisons were not statistically significant (**Figure 3H**). According to the ranking probability, RE was most likely to rank highest, followed by RE, AE, AE+RE, and HITT+RE (**Figure 4H**).

### 3.11 Effect of exercise on fatigue

Among 19 studies evaluating fatigue (FACT-F), four directly compared AE versus control groups, five compared RE versus control groups, three compared HIIT versus control groups, and nine compared AE+RE versus control groups.

Paired meta-analysis showed no significant difference between AE and control group (MD, -1.23; 95%CI-4.49, 2.04), RE and control group (MD, -0.66; 95%CI-2.94, 1.62), HITT and control group (MD, -0.90; 95%CI-9.66, 7.86), and AE+RE and control group (MD, 6.85; 95%CI-4.46, 18.15).

Since the node segmentation analysis showed no significant inconsistency (all p-values>0.05), a network meta-analysis of the consistency model was performed. Both the pairwise analysis and network analysis based on the consistency model revealed no significant differences between the AE, AE+RE, HIIT, HITT+RE, LMI, RE, and control groups (**Figure 3I**). The ranking of exercise interventions indicated that LMI was the most likely to be the most effective form of exercise for fatigue (FACT-F) (**Figure 4I**).

### 3.12 Effect of exercise on 400 metre walk test

Among the 8 studies that directly compared AE+RE with control group in 400 m walk test, the results of the meta-analysis showed that AE+RE was better than control group (MD, -10.82; 95%CI, -19.64, -2.00).

Since the closed-loop was not constructed in the network meta-analysis of the 400-meter walk test, node splitting analysis was not applicable. The consistency model-based network meta-analysis revealed that the AE+RE intervention outperformed the control (MD: -11.04; 95% CI: -21.25; -0.82) (**Figure 3J**). The ranking of exercise interventions indicated that RE was most likely to rank highest, followed by AE+RE (**Figure 4J**).

### 3.13 Effect of exercise on 6-min walk test (m)

Among five studies evaluating the 6-minute walk test (m), three directly compared AE+RE versus control group efficacy. A meta-analysis of these studies showed AE+RE was superior to control group (Mean Difference, 51.10; 95% CI 16.36, 85.84).

Since the closed-loop design was not established in the network meta-analysis of the 400-meter walk test, node splitting analysis was not applicable. The consistency model-based network meta-analysis revealed that AE+RE intervention outperformed the control group (MD 51.10; 95%CI 16.04–86.16), RE intervention was superior (MD 66.50; 95%CI 3.61–129.39), and AE intervention showed the greatest benefit (MD 78.80; 95%CI 21.92–135.68) (**Figure 3K**). The exercise intervention rankings indicated that AE was most likely to rank highest, followed by RE, AE+RE, and HIIT (**Figure 4K**).

### 3.14 Effect of exercise on VO2peak

Among 10 studies evaluating VO2peak, 2 directly compared AE versus control group efficacy, 2 compared RE versus control group efficacy, 3 compared HIIT versus control group efficacy, and 3 compared AE+RE versus control group efficacy.

Paired meta-analysis revealed that AE showed superiority over the control group (MD, 1.89; 95%CI 0.23, 3.54), while AE+RE demonstrated greater efficacy (MD, 1.96; 95%CI 0.20, 3.72). No significant differences were observed between RE and the control group (MD, 2.17; 95%CI-0.62, 4.95) or between HIIT and the control group (MD, 1.86; 95%CI-0.36, 4.08).

Since the node segmentation analysis showed no significant inconsistency (all p-values>0.05), a network meta-analysis of the consistency model was performed. The results demonstrated that AE alone outperformed the control in improving VO2peak (MD, 1.80; 95%CI 0.16, 3.44), AE+RE showed greater improvement than the control (MD, 2.03; 95%CI 0.45, 3.61), HITT had a more significant effect (MD, 1.83; 95%CI 0.15, 3.50), and RE exhibited the strongest improvement (MD, 2.43; 95%CI 0.63, 4.24) (**Figure 3L**). The ranking of all exercise interventions in terms of VO2peak improvement (best to worst) was: RE, AE+RE, HIIT, AE (**Figure 4L**).

## 4 Discussion

This study conducted a rigorous systematic review and network Meta-analysis to comprehensively evaluate the effects of different exercise interventions on health-related quality of life (HRQoL) and multidimensional physical function in prostate cancer patients undergoing androgen deprivation therapy (ADT). The research incorporated 41 randomized controlled trials (RCTs) involving 3, 588 patients, constructing a comprehensive evidence network that included aerobic exercise (AE), resistance training (RE), combined AE+RE, high-intensity interval training (HIIT) and its combinations, as well as low-intensity exercise (LMI). The analysis revealed that exercise interventions significantly improved ADT-related side effects, with distinct advantage profiles observed across different outcome metrics. The following sections will provide in-depth interpretation and discussion of the study’s findings from multiple perspectives, including key discoveries, multi-level mechanisms of action, clinical translation implications, research limitations, and future directions.

### 4.1 The main findings of the research are explained and analyzed in depth

#### 4.1.1 Differential improvement patterns of health-related quality of life

The health related quality of life (HRQoL) has become the key indicator of the quality of treatment experiences and general wellness of cancer patients. This paper marked the initial web-based meta-analysis to compare the impact of different exercise regimens on the HRQoL in patients who are receiving androgen deprivation therapy (ADT). The findings revealed that aerobic exercise and resistance training (AE +RE) is characterized by the highest improvement in the disease-specific quality of life scale (FACT-P) (Mean Difference [MD] = 7.96, 95% CI: 2.9512.96) and is ranked number 1 in the SUCRA ranking system. The result has a significant clinical implication because the FACT-P scale concentrates on prostate cancer-specific symptoms (pain, urinary and sexual difficulties), and its effects on the quality of life. The higher quality of AE+RE can be attributed to the fact that the former uplifts the overall vitality and psychological well-being through the cardiovascular functionality and alleviating of fatigue (28–30) whereas the latter directly raises the physiological performance and self-confidence due to the strength of muscles and improved body image (31, 32) Both physical and psychological improvements are positive to improve and this builds the basis of improved HRQoL.

Contrarily, even though AE+RE also scored high in general quality of life relevant scales (e.g., EORTC QLQ-C30 and SF-36), the level of effect size was not statistically significant. This difference could be attributed to the fact that the scales were different in focus. EORTC QLQ-C30 and SF-36 mostly examine general health situation and general simple daily living functions, although most of the distress caused by ADT is disease-specific. Also, not doing studies using such scales might have a small statistical power due to their relatively small number. Such results also indicate that the studies in the future must integrate both general and disease-specific scales in order to identify the comprehensive benefits of exercise interventions.

It is important to note that single-mode exercise (e.g., isolated AE or RE) did not exhibit any significant improvement of HRQoL. This indicates that multidimensional issues of ADT treatment can be better tackled with multi-target exercise programs than uni-modality training programs. This result is consistent with the general opinion regarding the use of integrated exercise regiments as suggested in the International Guidelines to Cancer Exercise (25, 33, 34).

#### 4.1.2 Targeted regulation of body composition: management of the paradox of muscle and fat

Among the most remarkable negative consequences of ADT, there is the deleterious change in body composition: the decrease in lean body mass (LBM) and the growth of the fat mass (BFM) and fat percentage (BFR). This study demonstrates how the various exercise programs can reverse this paradox.

Among SUCRA exercises, resistance training (RE) showed unsurpassed excellence with regard to lean body mass (MD = 2.79 kg, 95% CI: 0.6806.31) and was ranked number one. This is directly associated with the fact that resistance training has such a strong effect on the synthesis of muscle proteins. Low-testosterone state induced by ADT puts the patients into a muscle catabolic dominance condition, and RE stimulates anabolic signal pathways mTOR by mechanical load, which is effective in counteracting muscle atrophy (12, 35, 36). It is worth noting that AE+RE had a small positive influence on LBM (MD = 0.89 kg), but its effectiveness was much lower compared to that of RE. It could be attributed to the fact that the growth factors in combined training may be balanced by the energy spent on the aerobic training.

Comparing to the management of body fat, aerobic exercise (AE) has the largest impact on the body fat mass (BFM: MD = -3.30 kg), whereas high-intensity interval training and resistance training (HIIT+RE) takes the first place in terms of reducing the body fat percentage (BFR). The traditional fat-loss strategy is AE maximizing its consumption of fat as the energy source by means of intense moderate-intensity work. HIIT+RE can be more effective than regular trainings, though, in terms of creating a more pronounced after burn effect through post-exercise excess oxygen consumption (EPOC) and maintaining or even raising the ratio body composition through the use of resistance training (37–39). These results offer specific clinical conclusions: in those patients with major muscle loss, the resistance training is to be accentuated, in those with a high exposure to the risk of obesity, the stress is to be put on aerobic activity or on the elements of high-intensity interval training.

#### 4.1.3 Muscle strength and movement ability: from basic strength to comprehensive function

Strength of the muscles, especially lower limbs is a vital factor in ensuring independence and prevention of falls among the elderly. The research supports the hypothesis that resistance exercise (RE) is the most effective exercise to improve the strength of the upper limb (mean difference [MD] = 6.71 kg) and lower limb strength (MD = 38.03 kg). ADT-induced muscle loss in the lower limbs (e.g., iliopsoas and quadriceps) specifically causes gait instability and reduced mobility (40, 41). Specific resistance training is also capable of reversing the process with mechanisms that also include more muscle cross-sectional area as well as increased neuromuscular adaptability.

Regarding exercise capacity, AE +RE showed best results in 400-meter walking test (MD = -11.04 seconds) and 6-minute walking test (MD = 51.10 meters). These tests comprehensively reflect cardiorespiratory endurance, muscular endurance, and patients’ daily functional capacity. The high efficiency of the AE+RE makes it clear that the AE component will increase the efficiency of the cardiovascular system and the metabolism of energy, and the RE component will increase the functional reserve of the muscle and the tolerance to fatigability. The interesting conclusion was that resistance exercise (RE) was the best in terms of its ability to enhance maximal oxygen uptake (VO 2 peak) (MD = 2.43 ml/kg/min), outdoing aerobic exercise. This undermines classical ideas, meaning that an augmented mass of muscle itself will be able to augment the utilization of oxygen efficiency and in the long run, the cardiorespiratory fitness (42, 43).

Previous studies have shown that resistance training can improve mitochondrial density and oxidative capacity in skeletal muscle, independent of changes in muscle mass (44, 45). The enhanced muscle oxidative capacity following RE may explain its superiority in improving VO2peak in ADT patients, who experience significant muscle atrophy and mitochondrial dysfunction.

It should be noted that while RE did not show statistically significant differences compared to control in the paired meta-analysis, the network meta-analysis (which incorporates both direct and indirect evidence across all interventions) revealed RE as the highest-ranked intervention for VO2peak improvement. This discrepancy arises because the network analysis leverages information from the entire evidence network, providing more precise estimates than pairwise comparisons alone.

#### 4.1.4 Special considerations for fatigue management: balance between intensity and tolerance

One of the most widespread and distressing symptoms in the patients of ADT is cancer-related fatigue. Although there were no statistically significant differences in different exercise interventions with the control group in this study, the low-intensity exercise (LMI) was ranked on the first place in the SUCRA scale. The clinical implication of this finding(s) is significant. In the severely fatigued patients, the abrupt change to moderate to high-intensity exercises can lead to poor adherence since they experience discomfort and even worsen their fatigue. LMI (e.g., Tai Chi and Qigong) may be used as a moderately tolerable intervention to regulate the functioning of the autonomic nervous system, decrease inflammation, and enhance sleeping quality, which is a potentially widely acceptable and effective means of entry-level intervention with these patients (46, 47). This leads to placing exercise prescriptions on the principle of individualization and progression, which is capable of dynamically changing the intensity of exercise regarding the level of fatigue and physical condition of the patients.

### 4.2 Discussion on the multi-level and cross-system mechanism of action

Exercise intervention does not lead to enhancement of side effects of ADT, but a combination of many systems, i.e., hormone, metabolism, inflammation, neuropsychology, etc.

#### 4.2.1 Hormone and the reprogramming of molecular metabolic pathways

The principle working mechanism of ADT consists in the development of a low-androgen metabolic environment which directly inhibits anabolic secretion and promotes the catabolic reactions. The strongest anabolic stimulus, resistance training (RE) acts like the above mechanisms: ① Resistance training promotes muscle regeneration and hypertrophy by activating satellite cells and atrophy-related myogenic regulators (i.e. MyoD, myogenin); ②ecycle The most important signalling pathway: Resistance training activates insulin-like growth factor-1 (IGF-1) and its downstream pathway PI3K/Akt/mTOR, a direct start of muscle protein synthesis; ③ Concom This molecular-level reprogramming is the core of the RE in order to reverse muscle loss.

These mechanisms are supported by evidence showing that mechanical loading during RE activates the Akt/mTOR pathway and/or promotes satellite cell proliferation (48, 49).

Conversely, the control of energy metabolism is mainly controlled by aerobic exercise (AE). It stimulates insulin sensitivity and fatty acid oxidation by activating the translocation of skeletal muscle glucose transporter GLUT4 and enhancing insulin sensitivity and lipid metabolism through the activation of the AMPK pathway (50, 51). This process is essential to address ADT-related metabolic syndrome, fat accumulation in the body, and cardiovascular risk (9, 16, 52).

#### 4.2.2 Systemic inflammation and immune regulation

Androgen Deprivation Therapy (ADT), fatigue, muscle atrophy, and deterioration in the quality of life have a common pathophysiological mechanism of chronic low-grade inflammation. ADT per se triggers the production of pro-inflammatory cytokines (e.g., TNF- a, IL-6). It has been established that exercise can be used as an anti-inflammatory intervention. Regular exercise will be able to: 1) induce skeletal muscle to release IL-6, thus stimulating the formation of anti-inflammatory factors (e.g., IL-10, IL-1ra) and inhibiting TNF-a; 2) reduce visceral fat tissue (the main source of inflammatory factors); 3) cause the redistribution and functional enhancement of immune cells (e.g., natural killer cells, NK cells) (53, 54). This anti-inflammatory property not only reduce fatigue but also creates a more favorable microenvironment to muscle maintenance.

The anti-inflammatory effects of exercise have been consistently demonstrated in prostate cancer patients undergoing ADT, with regular exercise training shown to reduce circulating levels of C-reactive protein, IL-6, and TNF-α while increasing IL-10 (55).

#### 4.2.3 Activation of neuropsychological and behavioral mechanisms

It has been recognized that ADT is linked with the enhanced risk of depression, anxiety, and cognitive impairments (8, 21, 22, 56, 57). Physical exercise, especially more extensive medical methods such as AE + RE, may alter neuropsychological conditions in a variety of ways: ① By raising the level of brain-derived neurotrophic factor (BDNF) to stimulate neuroplasticity and hippocampal activity, which boost the mood and cognitive function;②Hypothalamic-pituitary-adrenal (HPA) axis regulation to decrease cortisol levels and mitigate psychological stress states; ③In group/supervised exercise, social interaction and support by the coach will be effective to increase self-efficacy, social belonging, and confidence in treatment in patients (25). These have psychological advantages, which are essential elements to the enhancement of health-related quality of life (HRQoL).

### 4.3 Clinical significance and practice transformation: constructing individualized exercise prescription framework

The results of the present research constitute a substantial level of evidence-based research in favor of the inclusion of exercise interventions into the normal treatment program in the clinical management of the ADT patients. On the basis of these findings, we offer a stratified and personalized system of exercise prescription: Basic Recommendation and Preferred Protocol: In the majority of ADT patients, aerobic exercise with resistance training (AE+RE) must be listed as the initial treatment to be applied. Based on the effective dosage of the studies included in the article, it is recommended to exercise with intensities of 2–3 times a week, an aerobic exercise with 65 percent-80 percent of maximal heart rate (HRmax) of 20–30 minutes, and resistance training with 60 percent-85 percent of 1-RM (6–12 repetitions per set, 2–4 sets). This is because the protocol does not only deal with declining health-related quality of life (HRQoL), but also muscle loss, increased body fat, and low cardiorespiratory function, among others, which means that the benefits can be achieved optimally (55, 58, 59).

Specific Improvement: The basic program must be tailored to meet the main concerns of the patient with the primary consideration being the gain of muscle mass and strength. In patients with a high level of sarcopenia or muscle weakness of severe severity, the active exercise (AE) + resistance (RE) model must increase the resistance (RE) option. This can include changing the frequency of resistance training to three days a week and giving preference to large-muscle and compound strength training.

Focus on losing fat and metabolic health: When patients are overweight/have metabolic syndrome, intensify aerobic exercise. Examples of high-intensity interval training (HIIT), including brief, peaking sprints with a coach present, can be considered to ensure maximum burning of fats and enhancement of metabolism.

The main idea is to treat severe fatigue: In patients with high ratings of fatigue caused by the cancer, initiate with low-intensity exercises (LMI) (i.e., Tai Chi, Qigong, or gentle yoga) with the purpose to form exercise regimes, and enhance sleep and mood (45). When the fatigue has been avoided, slowly move to moderate-high intensity exercises.

Model of Implementation and Support System: To achieve the long-term compliance, one can suggest the following phased support model: In the first stage (e.g., the first 12 weeks), employing the supervised group training is better to ensure that people can perform the movements correctly and safely, but use the group dynamics to enhance interest. The intermediate period must then move to a blend of the supervised sessions and the home-based training. Self-sustained active lifestyles should be embraced by the patients with regard to long-lasting maintenance processes. Also, the outcomes can be further condensed by combining behavioral change strategies (e.g., setting goals, self-monitoring) and digital health interventions (e.g., fitness apps).

According to the integrative medicine point of view, exercise intervention must not exist on its own but be part of a multidisciplinary team (MDT) approach to the management of prostate cancer. Oncologists, rehabilitation physicians, physical therapists, dietitians, and psychologists have to review the exercise prescriptions and integrate them with the nutritional support (e.g., ensuring that the patient receives enough protein to build muscle mass), psychological counseling, and the regular medical treatment, and optimize patient results in the long-term (25, 26).

### 4.4 Limitations and future directions

Despite the robust evidence generated by this study, several limitations should be acknowledged, which also point to important directions for future research.

Methodological heterogeneity and risk of bias: There was a high level of extremes of heterogeneity in parameters of exercise intervention (frequency, intensity, duration and mode of delivery [supervised vs. unsupervised]) in the included studies that could affect the accuracy of results. In addition, participant blinding in exercise studies is cumbersome, and not all studies satisfactorily reveal concealment of the allocation, which can create a risk of introducing performance and measurement bias. In future studies, the exercise protocol reporting should be standardized (at least using guidelines such as TIDieR) and the blinding and randomization methodology should be described in greater detail to improve the quality of evidence.

Absence of long-term efficacy and safety data: The majority of studies have followed a time frame of intervention or only short term follow-ups of the interventions providing no information on the impact of exercise interventions on long outcomes of survival rates, incidence of cardiovascular events, and risk of fractures in ADT patients. Since ADT is a long-term or even life-long treatment, large-scale, long-term (≥5 years) randomised controlled trial or pragmatic clinical trial are urgently required to demonstrate the long-term effects of exercise in terms of its benefits and safety. Additionally, while exercise may transiently increase circulating testosterone levels, current evidence suggests that regular moderate-intensity exercise does not significantly elevate baseline testosterone in ADT patients and is considered safe in clinical practice (60, 61).

Inadequate exploration of mechanisms: Clinical endpoint is used as the main feature of the current research, without any systematic monitoring of possible biomarkers (e.g., sex hormone levels, inflammatory factor profiles, myostatin and metabolomic markers). The future mechanistic research must be integrated into the program of clinical trials whereby blood and muscle biopsy samples are used to clarify the biological mechanisms that underlie the benefits associated with exercise; thus, forming the basis of the biomarker-based precision sports medicine.

Individual Differences and Accurate Exercise Prescription: The current literature did not find subgroups of patients (e.g., by age, and baseline fitness, ADT duration, genetic backgrounds, etc.) that are better suited to particular exercise programs. Future research is needed to alter the factors and come up with predictor models to allow personalized prescriptions. Besides, more efforts are supposed to be incorporated on patients of old age, very advanced stage of stressful illness, or possessing multiple comorbidities since their needs might be more complicated. Recent studies have also identified genetic polymorphisms that may modulate individual responses to exercise interventions in prostate cancer survivors. For instance, the APOE genotype has been shown to influence the effects of strength training on cognitive outcomes in ADT-treated patients (62), while the ACTN3 R577X polymorphism may affect muscle mass and strength responses by regulating androgen receptor signaling (63).

Absence of scientific and health economics appraisal: The difficulty lies in the ability to implement effective exercise programs on a large scale in an actual health system and community.

## 5 Conclusion

This paper is a survey and a network system meta-analysis performed to explicitly assess the impact of various types of exercise on health-related quality of life (HRQoL) and physical functioning among patients with prostate cancer undergoing androgen deprivation therapy (ADT). The findings indicate that the exercise intervention is a good strategy of non-pharmacological management of side effects as a result of ADT. Combination of aerobic exercise with resistance training (AE +RE) was confirmed as the best treatment which enhanced the overall HRQoL and complete physical performance showing substantial joint advantages. Also, resistance exercise (RE) was found to exhibit excellent benefits in muscle degradation and improving upper/lower limb strength, whereas aerobic exercise (AE) was found to have better benefits in body fat and body composition. Low-intensity exercise (LMI) can be a better entry point among patients who experience severe fatigue symptoms.

The study contributes to the high-level evidence-based practice of coming up with individualized exercise regimens in the clinical practice. Our first-line recommendation is to focus on multilevel aerobic-resistance training to the majority of ADT patients, although the development of exercise elements is required to be adjusted to individual aspects (e.g., sarcopenia, obesity, or intense fatigue). The interventions regarding exercise are needed to be implemented into the routine prostate cancer management programs to reduce the adverse effects of treatments and increase the quality of life and the outcome measures in the long term. Future studies ought to be carried out to test the long term effectiveness, tests and estimation of safety, as well as creating accuracy workout prescriptions to different categories of patients.

## Data Availability

The original contributions presented in the study are included in the article/**Supplementary Material**, further inquiries can be directed to the corresponding author/s.
