# Supplementary material for: The impact of different exercise modalities on health-related quality of life in patients with prostate cancer undergoing androgen deprivation therapy: a systematic review and network meta-analysis
Source: Front Oncol. 2026 Aug 17;16:1821735. doi: 10.3389/fonc.2026.1821735 (PMC13526573; doi:10.3389/fonc.2026.1821735)
Supplement: Supplementary file 1 [file Table1.docx]

**Pubmed**

| Number | Search terms | Results |
| --- | --- | --- |
| #1 | ((("Prostatic Neoplasms"[Mesh]) OR (((((((((((((((((Prostate Neoplasms[Title/Abstract]) OR (Neoplasms, Prostate[Title/Abstract])) OR (Neoplasm, Prostate[Title/Abstract])) OR (Prostate Neoplasm[Title/Abstract])) OR (Neoplasms, Prostatic[Title/Abstract])) OR (Neoplasm, Prostatic[Title/Abstract])) OR (Prostatic Neoplasm[Title/Abstract])) OR (Prostate Cancer[Title/Abstract])) OR (Cancer, Prostate[Title/Abstract])) OR (Cancers, Prostate[Title/Abstract])) OR (Prostate Cancers[Title/Abstract])) OR (Cancer of the Prostate[Title/Abstract])) OR (Prostatic Cancer[Title/Abstract])) OR (Cancer, Prostatic[Title/Abstract])) OR (Cancers, Prostatic[Title/Abstract])) OR (Prostatic Cancers[Title/Abstract])) OR (Cancer of Prostate[Title/Abstract]))) | 187172 |
| #2 | ((((((((("Exercise"[Mesh]) OR (((((((((((((((((((((((((Exercises[Title/Abstract]) OR (Physical Activity[Title/Abstract])) OR (Activities, Physical[Title/Abstract])) OR (Activity, Physical[Title/Abstract])) OR (Physical Activities[Title/Abstract])) OR (Exercise, Physical[Title/Abstract])) OR (Exercises, Physical[Title/Abstract])) OR (Physical Exercise[Title/Abstract])) OR (Physical Exercises[Title/Abstract])) OR (Acute Exercise[Title/Abstract])) OR (Acute Exercises[Title/Abstract])) OR (Exercise, Acute[Title/Abstract])) OR (Exercises, Acute[Title/Abstract])) OR (Exercise, Isometric[Title/Abstract])) OR (Exercises, Isometric[Title/Abstract])) OR (Isometric Exercises[Title/Abstract])) OR (Isometric Exercise[Title/Abstract])) OR (Exercise, Aerobic[Title/Abstract])) OR (Aerobic Exercise[Title/Abstract])) OR (Aerobic Exercises[Title/Abstract])) OR (Exercises, Aerobic[Title/Abstract])) OR (Exercise Training[Title/Abstract])) OR (Exercise Trainings[Title/Abstract])) OR (Training, Exercise[Title/Abstract])) OR (Trainings, Exercise[Title/Abstract]))) OR ("Tai Ji"[Mesh])) OR ((((((((((Tai-ji[Title/Abstract]) OR (Tai Chi[Title/Abstract])) OR (Chi, Tai[Title/Abstract])) OR (Tai Chi Chuan[Title/Abstract])) OR (Taiji[Title/Abstract])) OR (Taijiquan[Title/Abstract])) OR (T'ai Chi[Title/Abstract])) OR (Tai Ji Quan[Title/Abstract])) OR (Ji Quan, Tai[Title/Abstract])) OR (Quan, Tai Ji[Title/Abstract]))) OR ("Yoga"[Mesh])) OR ("Exercise Movement Techniques"[Mesh])) OR ("Swimming"[Mesh])) OR (((((((Movement Techniques, Exercise[Title/Abstract]) OR (Exercise Movement Technics[Title/Abstract])) OR (Pilates-Based Exercises[Title/Abstract])) OR (Exercises, Pilates-Based[Title/Abstract])) OR (Pilates Based Exercises[Title/Abstract])) OR (Pilates Training[Title/Abstract])) OR (Training, Pilates[Title/Abstract]))) | 467632 |
| #3 | #1 AND #2 | 1693 |

**Embase**

| Number | Search terms | Results |
| --- | --- | --- |
| #1 | 'prostate tumor'/exp | 326264 |
| #2 | ('neoplasia of the prostate' OR 'neoplasm of the prostate' OR 'neoplastic prostate' OR 'neoplastic prostates' OR 'neoplastic prostatic' OR 'prostate gland tumor' OR 'prostate gland tumour' OR 'prostate neoplasia' OR 'prostate neoplasm' OR 'prostate tumorigenesis' OR 'prostate tumour' OR 'prostatic neoplasia' OR 'prostatic neoplasm' OR 'prostatic neoplasms' OR 'prostatic tumor' OR 'prostatic tumorigenesis' OR 'prostatic tumour' OR 'tumor of the prostate' OR 'tumorigenesis of the prostate' OR 'tumour of the prostate' OR 'prostate tumor'):ti,ab,kw | 18401 |
| #3 | #1 OR #2 | 327331 |
| #4 | 'kinesiotherapy'/exp OR 'exercise'/exp OR 'physical activity'/exp OR 'aerobic exercise'/exp OR 'tai chi'/exp OR 'yoga'/exp OR 'pilates'/exp OR 'swimming'/exp | 1056809 |
| #5 | ('corrective exercise' OR 'exercise movement techniques' OR 'exercise therapy' OR 'exercise treatment' OR 'kinesiotherapeutic intervention' OR 'kinesiotherapeutic method' OR 'kinesiotherapeutic procedure' OR 'kinesiotherapeutic technique' OR 'kinesiotherapeutical treatment' OR 'kinesitherapeutic exercises' OR 'kinesitherapeutic intervention' OR 'kinesitherapeutic method' OR 'kinesitherapeutic methodology' OR 'kinesitherapeutic procedure' OR 'kinesitherapeutic technique' OR 'kinesitherapeutic treatment' OR 'kinesitherapeutical treatment' OR 'kinesitherapy' OR 'SKTM (specialized kinesitherapeutic methodology)' OR 'specialised kinesitherapeutic methodology' OR 'specialized kinesitherapeutic methodology' OR 'therapeutic exercise' OR 'therapy, exercise' OR 'treatment, exercise' OR 'kinesiotherapy' OR 'activity, physical' OR 'physical activity' OR 'biometric exercise' OR 'effort' OR 'exercise capacity' OR 'exercise performance' OR 'exercise training' OR 'exertion' OR 'fitness training' OR 'fitness workout' OR 'physical conditioning, human' OR 'physical effort' OR 'physical exercise' OR 'physical exertion' OR 'physical work-out' OR 'physical workout' OR 'exercise' OR 'aerobic dance' OR 'aerobic dancing' OR 'aerobics' OR 'aerobics exercise' OR 'dancing, aerobic' OR 'exercise, aerobic' OR 'low impact aerobic exercise' OR 'low impact aerobics' OR 'step aerobics' OR 'aerobic exercise' OR 'Tai Chi Chuan' OR 'Tai Ji' OR 'Taiji quan' OR 'Taijiquan' OR 'Tai Chi' OR 'yogic meditation' OR 'yoga' OR 'pilates exercise' OR 'pilates' OR 'bathing beaches' OR 'swimmer' OR 'swimming test' OR 'swimming'):ti,ab,kw | 912509 |
| #6 | #4 OR #5 | 1416565 |
| #7 | #3 AND #6 | 7828 |

**Web of science**

| Number | Search terms | Results |
| --- | --- | --- |
| #1 | TS=(Prostatic Neoplasms) OR AB=(Prostate Neoplasms OR Neoplasms, Prostate OR Neoplasm, Prostate OR Prostate Neoplasm OR Neoplasms, Prostatic OR Neoplasm, Prostatic OR Prostatic Neoplasm OR Prostate Cancer OR Cancer, Prostate OR Cancers, Prostate OR Prostate Cancers OR Cancer of the Prostate OR Prostatic Cancer OR Cancer, Prostatic OR Cancers, Prostatic OR Prostatic Cancers OR Cancer of Prostate) | 140354 |
| #2 | TS=(Exercise OR Tai Ji OR Exercise Movement Techniques OR Swimming) OR AB=(Exercises OR Physical Activity OR Activities, Physical OR Activity, Physical OR Physical Activities OR Exercise, Physical OR Exercises, Physical OR Physical Exercise OR Physical Exercises OR Acute Exercise OR Acute Exercises OR Exercise, Acute OR Exercises, Acute OR Exercise, Isometric OR Exercises, Isometric OR Isometric Exercises OR Isometric Exercise OR Exercise, Aerobic OR Aerobic Exercise OR Aerobic Exercises OR Exercises, Aerobic OR Exercise Training OR Exercise Trainings OR Training, Exercise OR Trainings, Exercise OR Tai-ji OR Tai Chi OR Chi, Tai OR Tai Chi Chuan OR Taiji OR Taijiquan OR T'ai Chi OR Tai Ji Quan OR Ji Quan, Tai OR Quan, Tai Ji OR Movement Techniques, Exercise OR Exercise Movement Technics OR Pilates-Based Exercises OR Exercises, Pilates-Based OR Pilates Based Exercises OR Pilates Training OR Training, Pilates OR Swimming) | 679217 |
| #3 | #1 AND #2 | 2111 |

**Cochrane**

| Number | Search terms | Results |
| --- | --- | --- |
| #1 | MeSH descriptor: [Prostatic Neoplasms] explode all trees | 9276 |
| #2 | (‘Prostatic Cancer’ OR ‘Cancers, Prostatic’ OR ‘Cancer of Prostate’ OR ‘Cancer, Prostatic’ OR ‘Cancers, Prostate’ OR ‘Prostatic Cancers’ OR ‘Cancer of the Prostate’ OR ‘Prostate Cancer’ OR ‘ Cancer, Prostate’ OR ‘ Prostate Cancers’ OR ‘ Prostate Neoplasms’ OR ‘ Prostatic Neoplasm’ OR ‘ Neoplasms, Prostate’ OR ‘Neoplasms, Prostatic’ OR ‘Prostate Neoplasm’ OR ‘Neoplasm, Prostate’ OR ‘Neoplasm, Prostatic’):ti,ab,kw | 20340 |
| #3 | #1 OR #2 | 20340 |
| #4 | MeSH descriptor: [Exercise] explode all trees | 41880 |
| #5 | MeSH descriptor: [Tai Ji] explode all trees | 638 |
| #6 | MeSH descriptor: [Yoga] explode all trees | 1315 |
| #7 | MeSH descriptor: [Swimming] explode all trees | 674 |
| #8 | MeSH descriptor: [Exercise Movement Techniques] explode all trees | 3926 |
| #9 | (‘Activities, Physical’ OR ‘Physical Activities’ OR ‘Activity, Physical; Physical Activity; Exercises, Isometric; Exercise, Isometric; Isometric Exercises; Isometric Exercise; Exercise Trainings; Exercise Training; Trainings, Exercise; Training, Exercise; Exercise, Aerobic; Aerobic Exercise; Aerobic Exercises' OR 'Exercises, Aerobic' OR 'Acute Exercises' OR 'Acute Exercise' OR 'Exercise, Acute' OR 'Exercises, Acute' OR 'Physical Exercises' OR 'Exercise, Physical' OR 'Exercises, Physical' OR 'Exercises' OR 'Physical Exercise' OR 'Ji Quan, Tai' OR 'Taijiquan' OR 'Chi, Tai' OR 'Tai Chi Chuan' OR 'Tai Ji Quan' OR 'T'ai Chi' OR 'Tai-ji' OR 'Tai Chi' OR 'Quan, Tai Ji' OR 'Taiji' OR 'Pilates Based Exercises' OR ' Exercises, Pilates-Based' OR ' Pilates Training' OR ' Pilates-Based Exercises' OR ' Training, Pilates’):ti,ab,kw | 11300 |
| #10 | #4 OR #5 OR #6 OR #7 OR #8 OR #9 | 130787 |
| #11 | #3 AND #10 | 801 |

**CINAHL**

| Number | Search terms | Results |
| --- | --- | --- |
| S1 | [SU (Prostatic Neoplasms) OR XB ("Prostate Neoplasms" OR "Neoplasms, Prostate" OR "Neoplasm, Prostate" OR "Prostate Neoplasm" OR "Neoplasms, Prostatic" OR "Neoplasm, Prostatic" OR "Prostatic Neoplasm" OR "Prostate Cancer" OR "Cancer, Prostate" OR "Cancers, Prostate" OR "Prostate Cancers" OR "Cancer of the Prostate" OR "Prostatic Cancer" OR "Cancer, Prostatic" OR "Cancers, Prostatic" OR "Prostatic Cancers" OR "Cancer of Prostate")](https://research.ebsco.com/search/results?db=ccm&expanders=concept&limiters=None&searchMode=boolean&sort=relevance&sqId=sq:7ad82335-cb7d-4b4d-be36-d580f9203041&userDirectAction=true) | 48904 |
| S2 | ([SU (exercise) OR XB ("Exercises" OR "Physical Activity" OR "Activities, Physical" OR "Activity, Physical" OR "Physical Activities" OR "Exercise, Physical" OR "Exercises, Physical" OR "Physical Exercise" OR "Physical Exercises" OR "Acute Exercise" OR "Acute Exercises" OR "Exercise, Acute" OR "Exercises, Acute" OR "Exercise, Isometric" OR "Exercises, Isometric" OR "Isometric Exercises" OR "Isometric Exercise" OR "Exercise, Aerobic" OR "Aerobic Exercise" OR "Aerobic Exercises" OR "Exercises, Aerobic" OR "Exercise Training" OR "Exercise Trainings" OR "Training, Exercise" OR "Trainings, Exercise")](https://research.ebsco.com/search/results?db=ccm&expanders=concept&limiters=None&searchMode=boolean&sort=relevance&sqId=sq:d365a617-e003-4963-b8ca-20bc5a02a6b2&userDirectAction=true) | 237982 |
| S3 | [SU (tai ji) OR XB ("Tai-ji" OR "Tai Chi" OR "Chi, Tai" OR "Tai Chi Chuan" OR "Taiji" OR "Taijiquan" OR "T'ai Chi" OR "Tai Ji Quan" OR "Ji Quan, Tai" OR "Quan, Tai Ji")](https://research.ebsco.com/search/results?db=ccm&expanders=concept&limiters=None&searchMode=boolean&sort=relevance&sqId=sq:fc187427-a14d-4254-907e-77cea6b30598&userDirectAction=true) | 2,346 |
| S4 | [SU (yoga) OR SU (exercise movement techniques) OR SU (Swimming) OR SU (Exercise Therapy) OR XB ("Movement Techniques, Exercise" OR "Exercise Movement Technics" OR "Pilates-Based Exercises" OR "Exercises, Pilates-Based" OR "Pilates Based Exercises" OR "Pilates Training" OR "Training, Pilates")](https://research.ebsco.com/search/results?db=ccm&expanders=concept&limiters=None&searchMode=boolean&sort=relevance&sqId=sq:0e596358-c570-40b9-a265-f7f11590feb2&userDirectAction=true) | 15,945 |
| S5 | S2 OR S3 OR S4 | 250702 |
| S6 | S1 AND S5 | 980 |

**知网 (China National Knowledge Infrastructure , CNKI)**

| Number | Search terms | Results |
| --- | --- | --- |
| **#1** | (主题：前列腺肿瘤)OR(篇关摘：前列腺肿瘤+前列腺癌+前列腺癌症(精确)) | 47648 |
| **#2** | (主题：运动+太极+太极拳+瑜伽+身体锻炼+游泳)OR(篇关摘：身体活动+体育锻炼+体育运动+急性运动+运动，等长+静力锻炼+等距离运动+运动，需氧+需氧运动+运动训练+锻炼+练习运动技术(精确)) | 2106811 |
| **#3** | #1 AND #2 | 682 |

**万方 (Wanfang)**

| Number | Search terms | Results |
| --- | --- | --- |
| **#1** | (主题=前列腺肿瘤) OR 题名或关键词=(前列腺肿瘤 or 前列腺癌 or 前列腺癌症) | 157943 |
| **#2** | ((主题=(运动 or 太极 or 太极拳 or 瑜伽 or 身体锻炼 or 游泳)) OR 题名或关键词=(运动 or 太极 or 太极拳 or 瑜伽 or 身体锻炼 or 游泳 or 身体活动 or 体育锻炼 or 体育运动)) OR 题名或关键词=(急性运动 or 运动, 等长 or 静力锻炼 or 等距离运动 or 运动, 需氧 or 需氧运动 or 运动训练 or 锻炼 or 练习运动技术) | 1249920 |
| **#3** | #1 AND #2 | 858 |

**维普(Chinese Medical Journal Database, VIP )**

| Number | Search terms | Results |
| --- | --- | --- |
| **#1** | 前列腺肿瘤 OR 前列腺癌 OR 前列腺癌症 |  |
| **#2** | 运动 OR 太极 OR 太极拳 OR 瑜伽 OR 身体锻炼 OR 游泳 OR 身体活动 OR 体育锻炼 OR 体育运动 OR 急性运动 OR 运动, 等长 OR 静力锻炼 OR 等距离运动 OR 运动, 需氧 OR 需氧运动 OR 运动训练 OR 锻炼 OR 练习运动技术 |  |
| **#3** | #1 AND #2 | 221 |
